# Influence of Incisal Embrasures on Smile Aesthetics

**DOI:** 10.4317/jced.62981

**Published:** 2025-09-01

**Authors:** Maria Fermeiro, Liliana Gavinha Costa, Maria Conceição Manso, Mariano Herrero-Climent, Javier Gil, Aritza Brizuela-Velasco, Paulo Ribeiro

**Affiliations:** 1 FP-I3ID, Instituto de Investigação, Inovação e Desenvolvimento, Biomedical Health Sciences, Faculty of Health Sciences, Fernando Pessoa University, 4249-004 Porto, Portugal; 2 Rise- Health, Faculty of Health Sciences, Fernando Pessoa University, Fernando Pessoa Teaching and Culture Foundation, Rua Carlos da Maia, 296, 4200-150, Porto Portugal; 3 Porto Dental Institute. 4150-518 Porto. Portugal; 4 Bioengineering Institute of Technology. International University of Catalonia. Josep Trueta s/n. 08195 Barcelona. Spain; 5 DENS-ia Research Group, Faculty of Health Sciences, Miguel de Cervantes European University. Valladolid, Spain

## Abstract

**Background:**

The main aim was to investigate the influence of incisal embrasure variation on the perception of smile attractive-ness among laypeople and dentists in three countries: Portugal, France and Italy, and between gender, age and specialty of the clinicians.

**Material and Methods:**

Two initial facial photographs were used to create 6: three of a male model and three of a female model with different incisal embrasures. Participants: Portuguese (204 laypeo-ple/195 doctors). French (214 laypeople/199 doctors) and Italian (204 laypeople/210 doctors). The attractiveness of the image was assessed by completing a questionnaire (visual analog scale VAS: 0-10).

**Results:**

The results were analyzed with non-parametric comparisons (*p*<0. 05). Significant differences were found between physicians and laypersons in the three countries. The difference in perceived attractiveness was greatest in Portugal. The most attractive embrasure was round and the least attractive was rectangular. The perception of attractiveness did not differ according to medical specialty. Portuguese female doctors were more critical than male doctors; French laymen were more critical than others. Age had no effect on perceived attractiveness of the embrasure.

**Conclusions:**

It was found that there are differences in aesthetic perception between physicians and laypeople in the three countries. In Portugal, dentists are stricter in their aesthetic perception of the embrasure than laypeo-ple. This was not observed in Italy, while France showed an intermediate result between the two countries. Gen-der had some influence on the rating in Portugal and France, in contrast to age. The area in which the doctor practiced had no influence.

** Key words:**Smile aesthetics, Incisal embrasures, Aesthetic impact.

## Introduction

Creating the perfect smile has long been a goal of dentistry [1]. Nowadays, appearance plays an important role in social, romantic and economic life, as several authors have demonstrated [2]. An image that is pleasing to the human eye generates feelings of joy and positive thoughts about someone or oneself. This characteristic leads to increased self-esteem and self-confidence, which is due to our basic nature [3]. The expression of a person’s feelings and emotions is expressed through their face, with the smile being the most important part of facial ex-pression [4].

For this reason, the smile plays an important role in the balance and beauty of the face, as we tend to focus on it first, followed by the nose, eyes, hair and other features [5]. Therefore, aesthetics and dentistry complement each other, as their principles are very important in areas such as restorative dentistry, orthodontics, prosthodon-tics and periodontics.

According to the authors Francischone and Mondelli, the dentist has the aid of principles to make a smile harmo-nious and pleasant for the patient [6]. Polack and Mahn emphasize that the clinician should consider not only the individual characteristics of each tooth, but also the effect they have together with other oral characteristics, namely the size of the teeth, gingival exposure and tooth symmetry [7]. In addition to the macroesthetic vision, which is more broadly defined by the two previous authors, a microesthetic vision should be obtained, which is more precise and detailed, analyzing the individual aspect and anatomy of each tooth.

The understanding of the aesthetics of dental and facial features differs between dentists, especially orthodon-tists, and lay people (without scientific knowledge of dentistry). Therefore, it is important to emphasize that pa-tients’ goals do not always coincide with dentists’ aesthetic goals [8]. For this reason, they should consider the patient’s preferences, gender and age before performing an oral rehabilitation [9].

Despite the importance of dental aesthetics, little has been done to understand the contribution of smile compo-nents such as incisal embrasures, a macroesthetic parameter that must be taken into account [5,10]. According to the authors Foulger *et al*. and Rosenstiel and Rashid, there are not many studies in the literature that refer to aesthetic preferences regarding the different forms of these embrasures [10,11]. The incisal embrasures corre-spond to the inverted “V”-shaped angles between the maxillary anterior teeth: the central incisors (CI), the lateral incisors (LI) and the canines (C). This angle is determined by the position of the most apical interproximal contact point. This in turn moves progressively from anterior to posterior towards the gingiva due to the anatomy of these teeth. As a result, the embrasures between CI, CI and LI, LI and C increase in size and volume mesiodistally. Between the two central incisors, the incisal embrasure is smaller and narrower than the embrasure between the central incisor and the lateral incisor, which appears more asymmetrical and rounded. Between the LI and the C, the embrasure is more apical than the embrasure between the CI and LI, according to Pasukdee, *P* and Foulger *et al*. [5,10]. These authors also add that incisal embrasures are typically wider in young individuals due to the more rounded tooth angles. Incisal embrasures that are not well defined or even absent due to incisal wear result in teeth that are too uniform and have a more aged appearance. According to Baharav *et al*., restorative proce-dures or orthodontic treatments can be tailored to each patient’s dental anatomy based on aesthetic preferences regarding incisal embrasures at the final stage, which shows the importance of this type of study [13].

This study aims to evaluate the effects of variations in incisal embrasures morphology on the aesthetic perception and attractiveness of a smile by comparing the ratings of laypeople and dentists through quantitative and qualita-tive analyzes. The results can help to develop more efficient and effective treatment plans that meet both patient expectations and professional standards.

## Material and Methods

- Participants and data collection 

This cross-sectional observational study involved male and female lay people and dental professionals aged 18 years and older from three European countries: Portugal, Italy and France. The participants took part in an online survey.

The inclusion criteria for recruiting participants to this study were that they were over 18 years of age and volun-tarily completing the survey after being informed of the study objectives through an introductory statement at the beginning of the questionnaire. The only exclusion criterion was the elimination of any questionnaire that had not been fully completed.

A questionnaire was developed as a data collection tool, which was translated into Portuguese, Italian and French for use in the respective countries. Before completing the survey, participants were required to read and give their informed consent to ensure voluntary participation with the option to withdraw at any time. They were also informed that they could contact the researchers if they had any questions.

The questionnaire collected demographic information, including nationality, age and gender. In addition, the den-tists were asked to indicate their primary field of practice in order to identify possible differences in the perception of smile attractiveness between different professional fields.

The questionnaire was divided into 5 parts, with each part corresponding to an aesthetic parameter: Part 1: An-gulation of upper central incisors; Part 2: Variations in buccal corridor width; Part 3: Gingival exposure in the smile; Part 4: Variations in incisal edges; Part 5: Dental proportions.

Respondents were asked to look at the pictures in each part and rate them according to the degree of aesthetic attractiveness on a scale from 0 (not attractive) to 10 (very attractive). The questionnaire was only valid if com-pleted in full and respondents were asked to answer only once.

The questionnaire was anonymous, so that the confidentiality of the answers was guaranteed for the study. The data was stored in a secure location during the study and destroyed after publication.

If in doubt, participants could also contact a member of the research team using the contact information provided in the survey. This study was shared online via various platforms such as WhatsApp, email and Facebook.

Finally, participants were shown randomly arranged pictures of smiles and asked to rate their attractiveness using a Visual Analogue Scale (VAS) ranging from 0 (“not at all attractive”) to 10 (“very attractive”). The images used in the questionnaire were taken from two original facial photos taken in a professional photo studio.

Pictures

A color frontal facial photograph was taken with a Nikon D750 DSLR camera (Nikon, Tokyo, Japan) with ISO 125, an aperture of F22, a white balance of 5500K and a gray background, keeping a distance of two meters. A Go-dox SK300i studio flash was used for illumination. Prior to image acquisition, written consent was obtained from two volunteer models, a Caucasian male and a Caucasian female, for the use of their images.

The images used in the questionnaire were digitally edited from these original photos using Adobe Photoshop© (Adobe Inc., San Jose, CA, USA). Two perfectly symmetrical facial models were intentionally created by cropping the faces to show only the smile region, which was divided along the upper midline of the teeth. To achieve symmetry, the left side of the male model’s smile was duplicated, flipped horizontally and positioned on the right side. The same procedure was applied to the female model, but with the right side.

**Figure 1:**
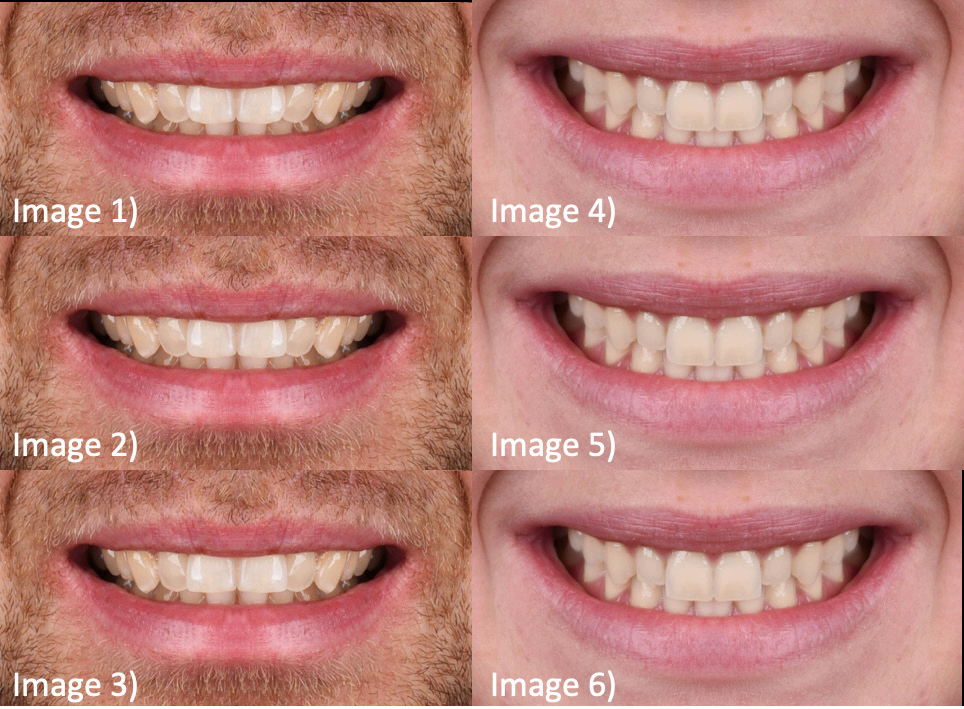
Digitally created images from the two initial photographs, using Adobe Photoshop©. Image 1: symmetrical smile with rounded incisal embrasures (Male); Image 2: symmetrical smile with semi-rounded incisal embrasures (Male); Image 3: sym-metrical smile with rectangular incisal embrasures (Male); Image 4: symmetrical smile with rounded incisal embrasures (Fe-male); Image 5: symmetrical smile with semi-rounded incisal embrasures (Female); Image 6: symmetrical smile with rectangular incisal embrasures (Female).

Brightness and contrast were adjusted to improve visibility. Finally, a total of six images were created — three of the female model and three of the male model. In each of these images, the incisal embrasures were digitally modified using the brush tool to create three variations (Fig. 1):

Rounded embrasures (more open)

Semi-rounded embrasures (medium)

Rectangular embrasures (more closed)

Ethical Considerations:

This study was approved by the ethical committee (Nº FCS/MED - 281/22) of the Universidade Fernando Pessoa. Participants were informed about the aim of the study and procedures and their consent was obtained before participating.

Data Analysis

The questionnaire data were compiled and exported to Excel for organization. Statistical analyses were then conducted using IBM© SPSS® Statistics v28.0 (IBM Corp., NY, USA). To assess significant differences in median Visual Analogue Scale (VAS) attractiveness scores between participant groups (laypeople vs. dentists, gender, age groups, and dental specialties), Mann–Whitney U tests were applied. Kruskal–Wallis tests were used to eval-uate differences across the three countries.Additionally, the attractiveness ratings of the six images were com-pared within each participant group using the Friedman test, followed by multiple comparisons with the Wilcoxon test and Bonferroni correction.

All analyses were performed considering a significance level of 5% (*p* < 0.05).

## Results

The study involved 1,261 adults: 641 lay people and 620 dental professionals, with a slight majority of male par-ticipants (52.4%). Only participants with Portuguese, French or Italian nationality were included in the analysis

Perception of attractiveness between dentists and laypeople

Analysis of the data ( Table 1) showed that Portugal had the largest difference in attractiveness perception be-tween laypeople and dentists, as four out of six images showed significant differences (*p*<0.05). Portuguese den-tists assigned lower values to the images than laypeople. The opposite was observed in France and Italy, where only one and two of the six images, respectively, showed significant differences in perceived attractiveness be-tween the two groups. In addition, dentists in these two countries assigned higher values to the images than laypeople. Overall, the dentists from the three countries perceived the images with round embrasures as more attractive than laypeople.

When comparing the perception of attractiveness of the 6 images between the dentists from the three countries, there were only significant differences in the perception of attractiveness for images 1 (male smile with round em-brasures) and 4 (female smile with round embrasures)(*p* = 0.011 and *p* = 0.014 respectively). Image 1 was per-ceived best by the Portuguese dentists, followed by the French and lastly by the Italian dentists. Image 4 was perceived best by the French dentists, followed by the Portuguese dentists and finally again by the Italian den-tists.

In general, the most attractive image for both genders was the one with the smile and round embrasures. However, the picture with the male smile was rated even better than the female one. The worst perceived image, common to all dentists, was image 6 with the rectangular embrasures in the female smile.

As for the laypeople, when comparing the perception of the attractiveness of the 6 images in Portugal, the image with the rounded embrasures in the male smile was the most attractive, while the rectangular embrasures in the female smile were the least attractive. For the other images, there was no significant difference in the perception of attractiveness. In contrast, French laypeople found four images equally attractive, with image 4 (round embra-sure in the female smile) being the most attractive. This differs from the other countries where image 1 (rounded embrasures in a male smile) was the most attractive. Although the rectangular embrasures in the female smile were rated the worst, there was no significant difference between them and the rectangular or semi-circular em-brasures in the male smile. and semi-circular in the female smile.

Finally, the Italian laypeople had a similar perception of attractiveness as the Italian dentists, with no significant differences observed for round and semi-rounded embrasures in images 1, 2, 4 and 5.

This study revealed that each image except image 5 (semi-rounded embrasures in female smile) was perceived differently by all laypeople in the three countries.

Perception of attractiveness between Dentist with and without professional focus on aesthetics.

As shown in  Table 2, there was no significant difference in attractiveness perception between these two groups.

Among dentists with expertise in aesthetic dentistry in the three countries, no significant differences were found in the perception of smile attractiveness. In contrast, among dentists without expertise in aesthetics, significant differences were only found for image 1 (rounded embrasures in male smile) (*p* = 0.010). This image was rated more positively by Portuguese and French dentists than by their Italian colleagues.

Portuguese dentists specializing in aesthetic dentistry rated image 1 (rounded embrasures in the male smile) as the most attractive, while image 4 (rounded embrasures in the female smile) and 6 (rectangular embrasures in the female smile) were the least attractive. In France, the images with the rounded embrasures (images 1 and 4) were rated the most attractive for both genders, while the image with the rectangular embrasures in the female smile (image 6) was rated the least attractive. Similar to the other countries, the male smile with rounded embra-sures (image 1) was rated as the most attractive in Italy, while the least attractive was the smile with rectangular embrasures in the male smile (image 3), which deviates slightly from the trend.

Among dentists, without a focus on aesthetics, the most attractive images were similar in Portugal and France: image 1 (rounded embrasures in male smile) and 4 (rounded embrasures in female smiles). The least attractive image were 6 for the Portuguese and 6 and 3 (rectangular embrasures in the female smile) for the French. In Italy, the most attractive images were 1 and 2 (rounded and semicircular embrasures in male smiles), while the least attractive image was once again image 6.

Perception of attractiveness between the sexes (women and men)

The analysis of  Table 3 shows that the perception of the attractiveness of the images is significantly different for women and men in Portugal and France. In Portugal, image 1 (round embrasures in the male smile) is perceived differently by male and female dentists (*p*=0.044), with male dentists rating it higher than female dentists. There is also a significant difference (*p*=0.005) between them for the semi rounded incisors in the female smile (Image 5), as well as for the rectangular embrasure in the female smile (Image 6) (*p*=0.038). It should be noted that the male dentists in Portugal had a higher perception of attractiveness than the female dentists, as they rated the images with higher scores overall. The laypeople in Portugal had a similar perception of attractiveness.

In France, on the other hand, there were no significant differences between male and female dentists, but there were differences between male and female laypeople, for images 1, 4, 5 and 6 (*p*=0.028; *p*=0.024; *p*=0.020; *p*=0.021, respectively). In contrast to Portugal and Italy, the male gender was more critical when it came to the perception of attractiveness.

Finally, in Italy, the perception of the attractiveness of the images, among both laypeople and dentists, did not differ by gender for any of the images, although the female gender was generally more critical, as in Portugal.

Perception of attractiveness between the under and over 45 age groups.

As shown in  Table 4, the perception of smile attractiveness did not differ significantly by age (with 45 years as the cut-off point) among either dentists or laypeople, except in Italy. In the Italian laypeople group, images 2 and 3 showed significant differences (*p* = 0.023 and *p* = 0.004, respectively), with participants over 45 years of age rating these images as more attractive than those 45 years or younger.

Overall, age had little influence on the perception of the attractiveness of the smile in the three countries.

## Discussion

The present cross-sectional study aimed to determine the effects of variations in incisal embrasures on the aes-thetic perception of the smile, as assessed by a group of participants who differed in terms of nationality, profes-sional background (laypeople or dentists), and demographic characteristics such as age and gender.

Six digitally modified images derived from two original sample images were used in this study. Chang *et al*. (2011) show that the gender of the models has a clinically significant influence on the aesthetic evaluation of the smile [14]. Therefore, in the present study,images of a male and a female model were used in the present study in order not to influence the attractiveness selection by the gender of the model.

Two symmetrical models — one male and one female — were created in which only the smile region was visible. Previous studies have shown that laypeople are more likely to recognize differences in smile attractiveness when presented with images that are limited to the oral region [15]. Krishnan *et al*. (2008) also suggested that using only the lower third of the face helps to minimize potential distractions in aesthetic perception [16]. In addition, focusing on the smile area facilitates the evaluation of fine details which may be less noticeable in full-face pho-tographs [10].

Although incisal embrasures play an important role in tooth morphology and thus in overall dental aesthetics, the topic is still poorly explored in the literature. To the best of our knowledge, there are no previous studies that have investigated the aesthetic perception of incisal embrasure variations taking into account evaluator-related factors, such as professional background or demographic characteristics.

Overall, the results showed that Portugal was the only country where dentists rated the images worse than lay-people ( Table 1), even when there was no significant difference in perception between the two groups. In Italy, on the other hand, dentists rated the images higher than laypeople. France was in the middle, with only two im-ages perceived better by laypeople than by doctors. Although the results from Portugal are consistent with those from a previous study with a similar design and objective [17], it is important to emphasize that the results of the Italian and French participants in our study differ from these results. This discrepancy could be due to cultural or regional differences in aesthetic perception as well as possible differences in clinical expectations or educational background of the raters.

The results show that, in general, rounded embrasures in male smile were rated as the most esthetically pleasing by dentists in all three countries. This is in contrast to the findings of Duarte M. *et al*. (2016) [17], in which lay-people, orthodontic patients, general dentists and orthodontists perceived semi-round embrasures as the most attractive.

In contrast, the perceptions of attractiveness among dentists in the three countries differed for only two images, indicating a certain homogeneity of opinions in this group for the remaining four. In addition, there were no significant differences in the aesthetic perception of embrasures between dental professionals with and without expertise in aesthetics and those without.

In relation to the six images evaluated images, the dentists with expertise in aesthetics from Portugal and France rated the rounded embrasures both male and female smiles as the most attractive, while the rectangular embrasures in female smiles were perceived as the least aesthetic. In the Italian sample, rounded embrasures in the male smile were also rated as most attractive in male smiles, while rectangular labia were rated as least attractive in male smiles, which differs slightly from the pattern observed in the other two countries.

These results are in line with previous studies indicating that rectangular incisal forms are generally perceived as less attractive compared to semi-rounded or rounded shapes [2]. This could be due to the fact that a more rounded contour on the mesial and distal angles of the anterior teeth improves the overall appearance, as the embrasures are directly influenced by the shape of the tooth itself.

The perceived attractiveness of the images, for both dentists and laypeople, did not differ according to the gender of the rater ( Table 3), with the exception of images 1, 5 and 6 for dentists in Portugal, where male doctors gave a higher rating than female doctors. This result is consistent with the results of a study by Flores-Mir C. *et al*. (2004), in which the female gender is more critical than the male gender. On the other hand, laypeople people in France contradicted the above-mentioned study [15]. The results of images 1, 4, 5 and 6 proved that there is a significant difference in perception between males and females, with the female gender giving a higher rating.

In addition, this study found that although pictures 2 and 3 in Italy showed significant differences in the perception of attractiveness by non-professionals, the age of the participants in the three countries had little overall in-fluence on their aesthetic perception.

In summary, physicians must not only create the most appropriate aesthetic treatment plan, including the selection of the embrasure type that best suits each individual practitioners must also take into account the cultural, social and personal preferences that shape patients’ aesthetic expectations. These preferences are often influenced by factors such as nationality, gender and even religious or societal norms. For treatment outcomes to be both effective and satisfactory, clear and empathetic communication between dentist and patient is essential to ensure mutual understanding and acceptance of the proposed outcome. With the increasing adoption of digital workflows in oral rehabilitation, clinicians now have the ability to perform diagnostic wax-ups where treatment goals are visualised through various design software and applications. This development also encourages the more frequent, efficient and cost-effective use of intraoral mock-ups, which allow multiple aesthetic factors — including incisal embrasure morphology —to be incorporated into the aesthetic treatment planning process.

In view of the complexity and subjectivity of aesthetic perception, this topic deserves further investigation in different population groups.

## Conclusions

In summary, the results of this study reveal remarkable differences in aesthetic perception related to the demographic and professional characteristics of the participants. Differences were found between dentists and lay-people in the three countries: In Portugal, dentists rated incisal embrasures more critically than laypeople, whereas this pattern was not observed in Italy. In France, the results were between the two countries. Gender showed some influence on aesthetic ratings in Portugal and France, while age did not appear to have a significant influence. The dentist’s specialty, regardless of whether it was related to aesthetics or not, also had no influence on perception.

However, despite these differences in perception between the groups, a general trend was observed: The rounded embrasure, particularly in the male smiles, was consistently rated as the most attractive, while the rectangular embrasure, particularly in female smiles, was perceived as the least attractive. These results emphasize the importance of considering factors related to the rater when evaluating smile aesthetics.

## Figures and Tables

**Table 1:** Cross-national comparison of perceived smile attractiveness between dental professionals and laypeople. (Values from 0 to 10, measured using a Visual Analogue Scale – VAS).

		Country							
		Portugal		France		Italy		p*** DDS	p*** Laypeople
		DDS	Laypeople	DDS	Laypeople	DDS	Laypeople		
Image 1 Rounded embrasures Male	average (SD)	6.5 (1.8)	6.73 (1.97)	6.54 (1.28)	6.1 (1.79)	6.23 (1.58)	5.85 (1.95)		
	Me (Q1-Q3)	7aA (5-8)	7aA (6-8)	7aAB (6-7)	6abB (5-7)	6aB (5.75-7)	6aB (5-7)	0.011	<0.001
	min-Max	1-9	0-10	2-10	0-10	0-10	0-10		
	p*	0.234		0.009		0.048			
Image 2 Semi-rounded embrasures Male	average (SD)	5.84 (1.55)	6.29 (1.61)	6.07 (1.4)	6 (1.78)	6.11 (1.47)	5.76 (1.87)		
	Me (Q1-Q3)	6b (5-7)	6bcA (6-7)	6b (5-7)	6abcAB(5-7)	6a (5-7)	6aB (5-7)	0.170	0.008
	min-Max	1-10	0-10	1-10	0-10	0-10	1-10		
	p*	0.001		0.827		0.043			
Image 3 Rectangular embrasures Male	average (SD)	5.46 (1.65)	6.24 (1.91)	5.74 (1.59)	5.83 (1.79)	5.72 (1.62)	5.48 (2.02)		
	Me (Q1-Q3)	5cd (5-7)	7bcA (5-8)	6c (5-7)	6bcB (5-7)	6bc (5-7)	5.5bcB (4-7)	0.263	<0.001
	min-Max	0-9	0-10	2-10	0-10	0-9	0-10		
	p*	<0.001		0.326		0.254			
Image 4 Rounded embrasures Female	average (SD)	6.21 (1.83)	6.42 (1.72)	6.42 (1.49)	6.14 (2.04)	6.01 (1.52)	5.86 (2.07)		
	Me (Q1-Q3)	7aAB (5-8)	7abA (6-7)	7aA (6-7)	6aB (5-8)	6aB (5-7)	6aB (4-7)	0.014	0.008
	min-Max	0-10	0-10	0-10	0-10	2-10	0-10		
	p*	0.328		0.192		0.731			
Image 5 Semi-rounded embrasures Female	average (SD)	5.73 (1.62)	6.11 (1.78)	5.96 (1.45)	5.89 (2.05)	5.86 (1.58)	5.69 (2.07)		
	Me (Q1-Q3)	6bc (5-7)	6bc (5-7)	6b (5-7)	6abc (5-7)	6ab (5-7)	6ab (4-7)	0.689	0.059
	min-Max	0-9	0-10	0-9	0-10	0-10	0-10		
	p*	0.006		0.854		0.592			
Image 6 Rectangular embrasures Female	average (SD)	5.4 (1.75)	6.04 (1.86)	5.44 (1.48)	5.65 (2.23)	5.59 (1.52)	5.24 (2.24)		
	Me (Q1-Q3)	6d (5-6)	6cA (5-7)	6c (5-6)	6cB (4-7)	6c (5-7)	5cB (4-7)	0.566	<0.001
	min-Max	0-9	0-10	0-9	0-10	1-9	0-10		
	p*	<0.001		0.135		0.173			
	p**	<0.001	<0.001	<0.001	<0.001	<0.001	<0.001		

1 *T. U Mann-Whitney test for comparison of groups; ** different letters identify significant differences in the median value of perceived attractiveness within the groups (“a” highest. “b” lowest. ... “e” lowest of all). according to Wilcoxon’s comparison test with Bonferroni’s correction.

**Table 2:** Statistical analysis of smile aesthetics perception results, comparing two groups of dentists based on whether or not they specialize in aesthetic dentistry (Values from 0 to 10, measured using a Visual Analogue Scale – VAS).

		Country							
		Portugal		France		Italy		p(Yes)***	p(No)***
		Areas of expertise with a focus on aesthetics		Areas of expertise with a focus on aesthetics		Areas of expertise with a focus on aesthetics			
		Yes	No	Yes	No	Yes	No		
	n	49	146	29	170	70	140		
Image 1 Rounded embrasure Male	average (SD)	6.45 (1.72)	6.51 (1.84)	6.45 (1.3)	6.56 (1.28)	6.2 (1.77)	6.24 (1.49)		
	Me (Q1-Q3)	7a (5-8)	7aA (5.75-8)	6a (6-7)	7aA (6-7)	7a (5-7)	6aB (6-7)	0.658	0.010
	min-Max	3-9	1-9	3-9	2-10	0-9	0-10		
	p*	0.682		0.633		0.554			
Image 2 Semi-rounded embrasure Male	average (SD)	6 (1.57)	5.79 (1.55)	6 (1.46)	6.08 (1.4)	5.96 (1.63)	6.19 (1.38)		
	Me (Q1-Q3)	6ab (5-7)	6b (5-7)	6b (5-7)	6b (5-7)	6ab (5-7)	6a (5-7)	0.972	0.093
	min-Max	1-9	2-10	3-8	1-10	0-8	0-10		
	p*	0.360		0.706		0.617			
Image 3 Rectangular embrasure Male	average (SD)	5.63 (1.87)	5.4 (1.57)	6 (1.56)	5.7 (1.59)	5.66 (1.67)	5.76 (1.6)		
	Me (Q1-Q3)	6ab (4-7)	5cd (5-7)	6bc (5-7)	6c (5-7)	6b (5-7)	6bc (5-7)	0.726	0.187
	min-Max	0-9	0-8	3-8	2-10	0-9	1-9		
	p*	0.297		0.292		0.841			
Image 4 Rounded embrasure Female	average (SD)	6.08 (1.77)	6.25 (1.85)	6.17 (1.71)	6.46 (1.45)	5.89 (1.54)	6.08 (1.52)		
	Me (Q1-Q3)	6b (5.5-7)	7a (5-8)	6a (5-7)	7a (6-7)	6ab (5-7)	6ab (5-7)	0.357	0.059
	min-Max	1-9	0-10	0-10	2-10	3-9	2-10		
	p*	0.517		0.211		0.329			
Image 5 Semi-rounded embrasure Female	average (SD)	5.63 (1.64)	5.77 (1.61)	5.76 (1.6)	5.99 (1.43)	5.74 (1.55)	5.92 (1.6)		
	Me (Q1-Q3)	6ab (5-7)	6bc (5-7)	6bc (5-6)	6b (5-7)	6ab (4.75-7)	6ab (5-7)	0.946	0.638
	min-Max	1-8	0-9	0-9	1-9	2-9	0-10		
	p*	0.735		0.349		0.386			
Image 6 Rectangular embrasure Female	average (SD)	5.47 (2.15)	5.38 (1.6)	5.1 (1.45)	5.49 (1.48)	5.71 (1.56)	5.52 (1.51)		
	Me (Q1-Q3)	6b(5-7)	6d(5-6)	5c(5-6)	6c(5-6)	6ab(5-7)	6c(5-7)	0.138	0.881
	min-Max	0-9	0-9	0-7	1-9	1-9	2-9		
	p*	0.322		0.255		0.427			
	p**	<0.001	<0.001	<0.001	<0.001	0.022	<0.001		

2 *T. U Mann-Whitney test for comparison of groups; A;B- different letters identify significant differences in the median value of perceived attractiveness between countries (“A” higher and “B” lower). according to Mann-Whitney comparison test after * Kruskal-Wallis test. **Friedman test for comparison of images (in each group of participants). a.b.c...and different letters identify significant differences in the median value of perceived attractiveness within groups (“a” highest. “b” lowest. ... “e” lowest of all). according to Wilcoxon’s comparison test with Bonferroni’s correction.

**Table 3:** Comparison of perceived image attractiveness by gender for each group (Values from 0 to 10, measured using a Visual Ana-logue Scale – VAS).

		Country											
		Portugal				France				Italy			
		DDS		Laypeople		DDS		Laypeople		DDS		Laypeople	
		M	F	M	F	M	F	M	F	M	F	M	F
	n	90	105	119	85	103	96	78	136	105	105	79	125
Image 1 Rounded embrasures Male	average (SD)	6.79 (1.68)	6.25 (1.87)	6.86 (1.82)	6.55 (2.16)	6.7 (0.84)	6.38 (1.61)	5.77 (1.76)	6.29 (1.79)	6.31 (1.51)	6.14 (1.66)	5.84 (1.96)	5.86 (1.95)
	Me (Q1-Q3)	7 (6-8)	7 (5-8)	7 (6-8)	7 (5-8)	7 (6-7)	7 (6-7)	6 (5-7)	7 (5-7.75)	6 (6-7)	6 (5-7)	6 (5-7)	6 (5-8)
	min-Max	02-9	01-9	02-10	0-10	04-9	02-10	0-10	02-10	0-10	0-10	0-10	01-10
	p	0.044		0.477		0.314		0.028		0.465		0.986	
Image 2 Semi-rounded embrasures Male	average (SD)	6.09 (1.4)	5.63 (1.65)	6.37 (1.37)	6.19 (1.91)	6.06 (1.14)	6.07 (1.64)	5.78 (1.77)	6.13 (1.78)	6.23 (1.51)	6 (1.43)	5.78 (1.93)	5.75 (1.83)
	Me (Q1-Q3)	6 (5-7)	6 (4-7)	6 (6-7)	6 (5-7)	6 (5-7)	6 (5-7)	6 (4.75-7)	7 (5-7)	6 (5.5-7)	6 (5-7)	6 (5-7)	6 (5-7)
	min-Max	03-9	01-10	02-9	0-10	03-9	01-10	0-10	01-10	0-10	0-9	01-10	01-10
	p	0.078		0.689		0.529		0.091		0.18		0.88	
Image 3 Rectangular embrasures Male	average (SD)	5.58 (1.61)	5.36 (1.68)	6.35 (1.72)	6.07 (2.14)	5.66 (1.45)	5.83 (1.73)	5.82 (1.86)	5.83 (1.75)	5.88 (1.84)	5.57 (1.35)	5.54 (2.03)	5.43 (2.01)
	Me (Q1-Q3)	5 (5-7)	5 (4-7)	7 (5-8)	6 (5-8)	5 (5-7)	6 (5-7)	6 (4.75-7)	6 (5-7)	6 (5-7)	5 (5-7)	5 (5-7)	6 (4-7)
	min-Max	0-9	0-8	01-10	0-10	02-9	02-10	0-10	0-10	0-9	02-8	0-10	01-10
	p	0.369		0.454		0.361		0.890		0.088		0.83	
Image 4 Rounded embrasuresFemale	average (SD)	6.47 (1.56)	5.99 (2.01)	6.4 (1.46)	6.44 (2.04)	6.42 (1.21)	6.42 (1.75)	5.68 (2.15)	6.41 (1.93)	6.11 (1.42)	5.91 (1.62)	5.7 (2.13)	5.97 (2.02)
	Me (Q1-Q3)	7 (6-8)	6 (5-7)	7 (6-7)	7 (5-8)	6 (6-7)	7 (6-7.75)	6 (4-7)	6.5 (5-8)	6 (5-7)	6 (5-7)	6 (4-7)	6 (5-7)
	min-Max	02-9	0-10	01-10	0-10	03-10	0-10	0-10	0-10	02-9	02-10	0-10	0-10
	p	0.114		0.378		0.523		0.024		0.484		0.434	
Image 5 Semi-rounded embrasures Female	average (SD)	6.09 (1.26)	5.43 (1.82)	6.06 (1.63)	6.18 (1.98)	6.06 (1.19)	5.85 (1.69)	5.44 (2.13)	6.15 (1.97)	6.01 (1.61)	5.71 (1.55)	5.78 (1.91)	5.62 (2.17)
	Me (Q1-Q3)	6 (6-7)	6 (5-7)	6 (5-7)	6 (5-7.5)	6 (6-7)	6 (5-7)	6 (4-7)	6 (5-7.75)	6 (5-7)	6 (5-7)	6 (5-7)	6 (4-7)
	min-Max	02-9	0-9	01-10	0-10	03-9	0-9	0-10	0-10	0-10	02-8	0-10	0-10
	p	0.005		0.423		0.558		0.020		0.237		0.691	
Image 6 Rectangular embrasures Female	average (SD)	5.77 (1.33)	5.09 (1.99)	6.15 (1.57)	5.89 (2.2)	5.44 (1.07)	5.44 (1.82)	5.22 (2.2)	5.9 (2.21)	5.74 (1.52)	5.43 (1.52)	5.38 (2.05)	5.14 (2.35)
	Me (Q1-Q3)	6 (5-6)	6 (4-6)	6 (6-7)	6 (5-7.5)	6 (5-6)	6 (4-7)	5.5 (3.7-7)	6 (4-7)	6 (5-7)	5 (4.5-7)	5 (4-7)	5 (4-7)
	min-Max	02-9	0-9	01-10	0-10	03-8	0-9	0-10	0-10	02-9	01-8	0-10	0-10
	p	0.038		0.545		0.586		0.021		0.223		0.662	

p - comparison in median self-perceived attractiveness by participants in each group in each country by the Mann-Whitney test and comparison of preference per side by the chi-square test (bold values indicate significant differences in the attractiveness rating/ preference attributed by group in the same country)

**Table 4:** Comparison of perceived attractiveness using 45 years as the cutoff. (Values from 0 to 10, measured using a Visual Analogue Scale – VAS).

		Country											
		Portugal				France				Italy			
		DDS		Laypeople		DDS		Laypeople		DDS		Laypeople	
		45	>45	45	>45	45	>45	45	>45	45	>45	45	>45
	n	142	53	167	37	142	57	159	55	129	81	154	50
Image 1 Rounded embrasure Male	average (SD)	6.46 (1.83)	6.6 (1.74)	6.82 (1.94)	6.32 (2.1)	6.54 (1.37)	6.56 (1.04)	6.13 (1.84)	6.02 (1.67)	6.22 (1.52)	6.25 (1.7)	5.69 (2.06)	6.32 (1.46)
	Me (Q1-Q3)	7 (5-8)	7 (6-8)	7 (6-8)	7 (5-8)	7 (6-7)	6 (6-7)	6 (5-7)	6 (5-7)	6 (5-7)	6 (6-7)	6 (4-7)	6 (5-8)
	min-Max	1-9	2-9	0-10	0-10	2-10	4-10	0-10	2-9	0-10	0-10	0-10	2-9
	p	0.657		0.203		0.324		0.616		0.825		0.071	
Image 2 Semi-rounded embrasure Male	average (SD)	5.8 (1.6)	5.96 (1.43)	6.29 (1.5)	6.32 (2.07)	6.09 (1.51)	6 (1.1)	5.99 (1.81)	6.04 (1.71)	6.12 (1.37)	6.11 (1.63)	5.59 (1.96)	6.3 (1.43)
	Me (Q1-Q3)	6 (5-7)	6 (5-7)	6 (6-7)	7 (5-8)	6 (5-7)	6 (5-7)	6 (5-7)	6 (5-7)	6 (5-7)	6 (5-7)	6 (4.75-7)	6 (5-8)
	min-Max	1-10	2-9	0-9	0-10	1-10	4-9	0-10	2-10	0-9	0-10	1-10	3-9
	p	0.395		0.569		0.320		1.000		0.952		0.023	
Image 3 Rectangular embrasure Male	average (SD)	5.46 (1.78)	5.47 (1.25)	6.26 (1.77)	6.14 (2.45)	5.85 (1.67)	5.49 (1.35)	5.77 (1.85)	5.98 (1.59)	5.64 (1.56)	5.86 (1.72)	5.23 (2.11)	6.22 (1.49)
	Me (Q1-Q3)	5 (4-7)	5 (5-6)	6 (5-8)	7 (5-8)	6 (5-7)	5 (5-6)	6 (5-7)	6 (5-7)	6 (5-7)	6 (5-7)	5 (4-7)	6 (5-7.25)
	min-Max	0-9	2-8	0-10	0-10	2-10	3-9	0-10	3-9	1-9	0-9	0-10	3-10
	p	0.873		0.805		0.083		0.615		0.295		0.004	
Image 4 Rounded embrasure Female	average (SD)	6.13 (1.86)	6.43 (1.72)	6.46 (1.44)	6.24 (2.65)	6.39 (1.53)	6.47 (1.38)	6.23 (2.06)	5.89 (1.99)	5.96 (1.52)	6.1 (1.53)	5.8 (2.15)	6.06 (1.8)
	Me (Q1-Q3)	6 (5-7)	7 (5.5-8)	7 (6-7)	7 (5-8)	6 (6-7)	7 (6-7)	6 (5-8)	6 (4-8)	6 (5-7)	6 (5-7)	6 (4-7)	6 (5-7)
	min-Max	1-10	0-9	2-10	0-10	0-10	2-10	0-10	2-9	2-10	2-9	0-10	3-10
	p	0.303		0.544		0.689		0.249		0.384		0.685	
Image 5 Semi-rounded embrasure Female	average (SD)	5.65 (1.68)	5.96 (1.41)	6.12 (1.57)	6.05 (2.55)	5.87 (1.44)	6.18 (1.47)	5.95 (1.99)	5.71 (2.23)	5.76 (1.6)	6.02 (1.55)	5.61 (2.17)	5.92 (1.75)
	Me (Q1-Q3)	6 (5-7)	6 (5.5-7)	6 (5-7)	7 (5-8)	6 (5-7)	6 (6-7)	6 (5-7)	6 (4-8)	6 (5-7)	6 (5-7)	6 (4-7)	6 (4-7)
	min-Max	0-9	0-9	1-10	0-10	0-9	2-9	0-10	1-9	0-10	2-9	0-10	2-9
	p	0.434		0.286		0.182		0.635		0.199		0.581	
Image 6 Rectangular embrasure Female	average (SD)	5.32 (1.87)	5.6 (1.35)	6.03 (1.68)	6.11 (2.56)	5.35 (1.47)	5.65 (1.48)	5.63 (2.15)	5.73 (2.47)	5.49 (1.47)	5.74 (1.6)	5.07 (2.32)	5.74 (1.9)
	Me (Q1-Q3)	6 (4-7)	6 (5-6)	6 (5-7)	7 (5-8)	6 (5-6)	6 (5-6)	6 (4-7)	6 (4-8)	6 (5-6.5)	6 (5-7)	5 (4-7)	6 (4-7)
	min-Max	0-9	0-9	0-10	0-10	0-9	2-9	0-10	0-10	2-9	1-9	0-10	2-10
	p	0.660		0.238		0.344		0.634		0.200		0.130	

p - comparison in median self-perceived attractiveness by participants in each group in each country by the Mann-Whitney test and comparison of prefer-ence per side by the chi-square test (bold values indicate significant differences in the attractiveness rating/ preference attributed by group in the same coun-try);

## Data Availability

The datasets used and/or analyzed during the current study are available from the corresponding author.
